# The Treatment of Crural Hernia

**Published:** 1909-08

**Authors:** 


The Treatment of Crural Hernia.—Dr. J. Exalto reports from the clinic of Professor von Eiselsberg, Vienna (*Wiener Klin. Wochensch.,* April 22, 1909), 134 cases of crural hernia operated upon during the years 1901 to 1908. Of the patients 114 were females. In 72 the hernia was incarcerated. In 52 it was on the left side, in 77 on the right, and in 5 bilateral. There was no operative mortality in the non-strangulated cases. In 34 of 56 cases of intestinal incarceration the gut could be reduced, although in 10 suspicious areas were first covered with a few Lembert's sutures. In 17 the intestine had to be resected. Of the 72 cases of incarcerated rupture 19 died, a mortality of 26.4 per cent. The mortality in the 17 cases of primary intestinal resection was 47 per cent. In almost all the fatal cases death was due to peritonitis. —*International Journal of Surgery.*

